# Efficacy and safety of *trans*-fornix conjunctival retrobulbar block in a prospective randomized controlled trial

**DOI:** 10.1016/j.isci.2026.118026

**Published:** 2026-10-26

**Authors:** Ruibo Chen, Tao Xu, Chunming Chen, Kejun Liu, Guorong Shi, Chunyan Xi, Mengqing Yin, Ruirui Chen, Kai Hu, Bing Chen

**Affiliations:** 1 School of Medicine, Southeast University, Nanjing 210009, China; 2 Purui Eye Hospital, Changzhou 213003, China; 3 Changzhou Health Vocational Technology College, Changzhou 213002, China; 4 Xiamen Eye Center, Department of Ophthalmology, Affiliated to Xiamen University, Xiamen 361003, China; 5 Department of Ophthalmology, Nanjing Drum Tower Hospital, Affiliated Hospital of Medical School, Nanjing University, 321 Zhongshan Road, Nanjing 210008, China; 6 Guanghua Optometry Eye Hospital, Changzhou 213003, China; 7 Department of Ophthalmology, Liyang Hospital of Chinese Medicine, Liyang, China; 8 Guangming Eye Hospital, Gaoyou 225600, China

**Keywords:** retrobulbar block, *trans*-fornix conjunctival approach, vitreoretinal surgery, randomized controlled trial

## Abstract

Retrobulbar block is widely used for vitreoretinal surgery, but the conventional transcutaneous technique advances a sharp needle blindly through the lower eyelid. We evaluated a *trans*-fornix conjunctival approach in which a curved blunt-tip needle is advanced into the muscle cone under the operating microscope. In a two-center randomized trial, 196 patients were stratified by expected surgical duration and allocated to the transcutaneous or the *trans*-fornix technique, and pain was graded at predefined procedural stages. The *trans*-fornix approach reduced pain during anesthetic injection in both cohorts and during scleral indentation in longer procedures, and fewer patients required supplemental anesthesia. Ocular akinesia was better with the *trans*-fornix technique, and periorbital hemorrhage and ecchymosis occurred only in the transcutaneous group. These findings support the *trans*-fornix block as a practical and reproducible option for regional anesthesia in vitreoretinal surgery.

## Highlights

• A curved blunt-tip needle reaches the muscle cone through the conjunctival fornix
• The block is performed in one pass under the microscope without any incision
• Injection-related pain was lower than with the transcutaneous retrobulbar block
• Fewer patients needed supplemental anesthesia during scleral indentation

## Graphical abstract

**Figure:**
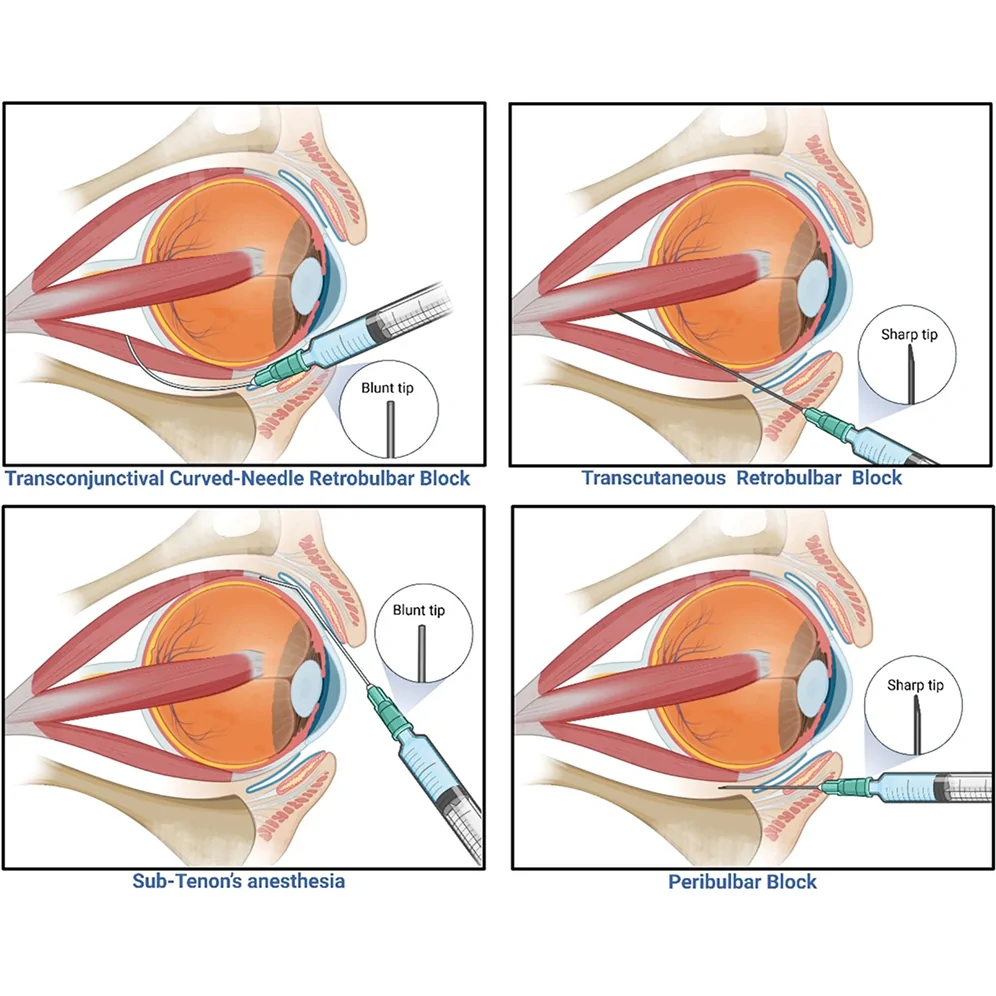

## Teaser

Anesthesiology; Health sciences; Medical specialty; Medicine; Ophthalmology

## Introduction

With the global shift toward ambulatory surgery, many intraocular procedures, including vitreoretinal surgery and glaucoma drainage device implantation, are routinely performed under retrobulbar block anesthesia. This approach is particularly suitable for cooperative patients, as well as elderly individuals with significant comorbidities who require prolonged operative times. Patients can be discharged immediately after a short observation. This saves medical resources and reduces the financial burden. In developing countries, particularly in resource-limited settings, the shortage of anesthesiologists and limited access to general anesthesia reinforce retrobulbar block as a cornerstone of ocular regional anesthesia. Therefore, retrobulbar block anesthesia remains the gold standard for ocular regional anesthesia. Traditional retrobulbar block administered via the transcutaneous approach is associated with vision-threatening and even life-threatening complications.^1^^,^^2^^,^^3^^,^^4^ Consequently, several alternative techniques, including peribulbar anesthesia and sub-Tenon’s anesthesia, have been developed and increasingly adopted.^5^^,^^6^

The ideal ophthalmic anesthesia should achieve profound analgesia with minimal complications. However, the alternative methods mentioned above have specific limitations that may restrict their universal application. In this study, we investigated a *trans*-fornix retrobulbar block using a custom-designed 27-gauge curved blunt-tip needle (Figures 1, 2, and 3). By comparing this technique with conventional transcutaneous retrobulbar block, this study aimed to compare visual analog scale (VAS)-based pain grades during anesthetic injection, incision creation, intraoperative manipulation, and scleral indentation for exposure of the vitreous base, as well as intraoperative and postoperative anesthesia-related complications.

**Figure 1:**
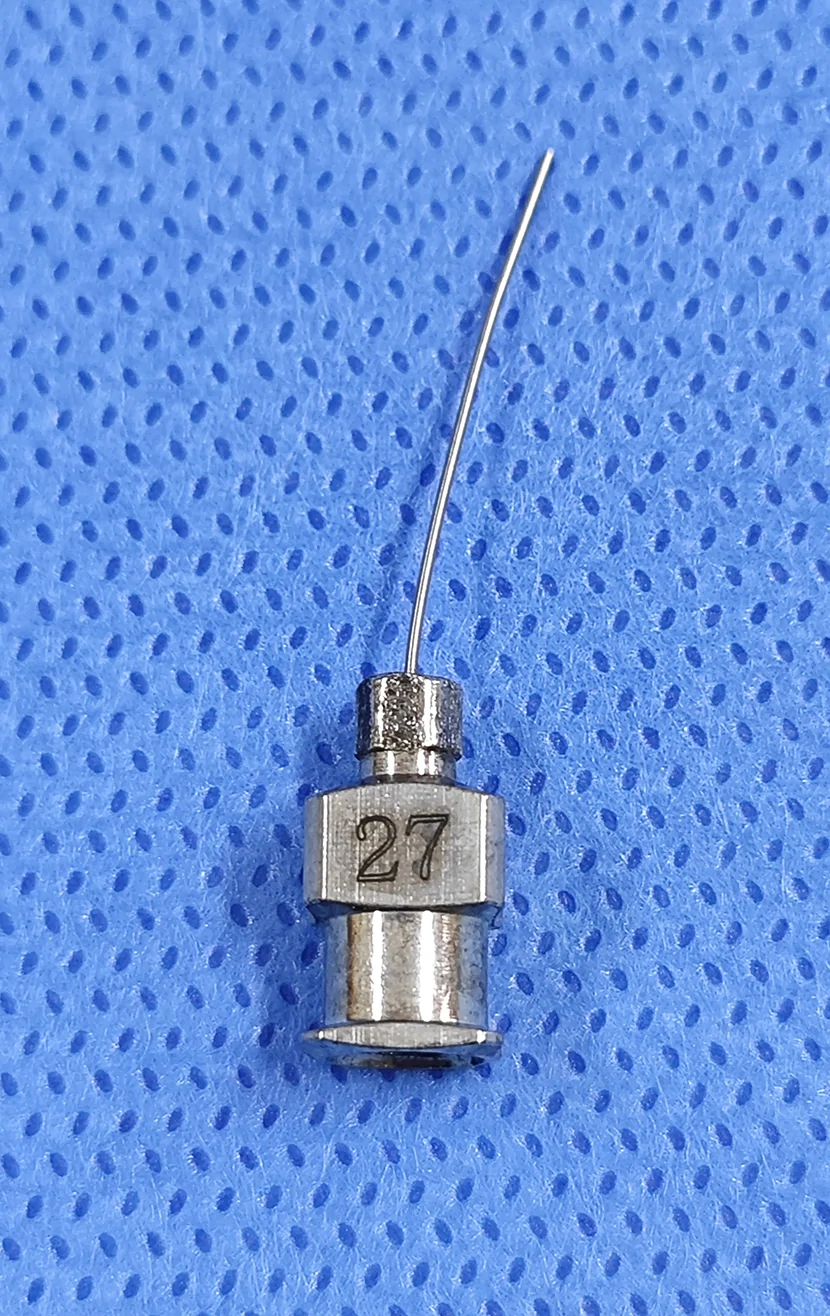
The curved blunt-tip needle used in the experimental group The image displays the 27-gauge, 20-mm-long stainless steel needle manufactured by Nanjing ChuangKeDa Medical Instrument Co., Ltd. (Jiangsu, China).

**Figure 2:**
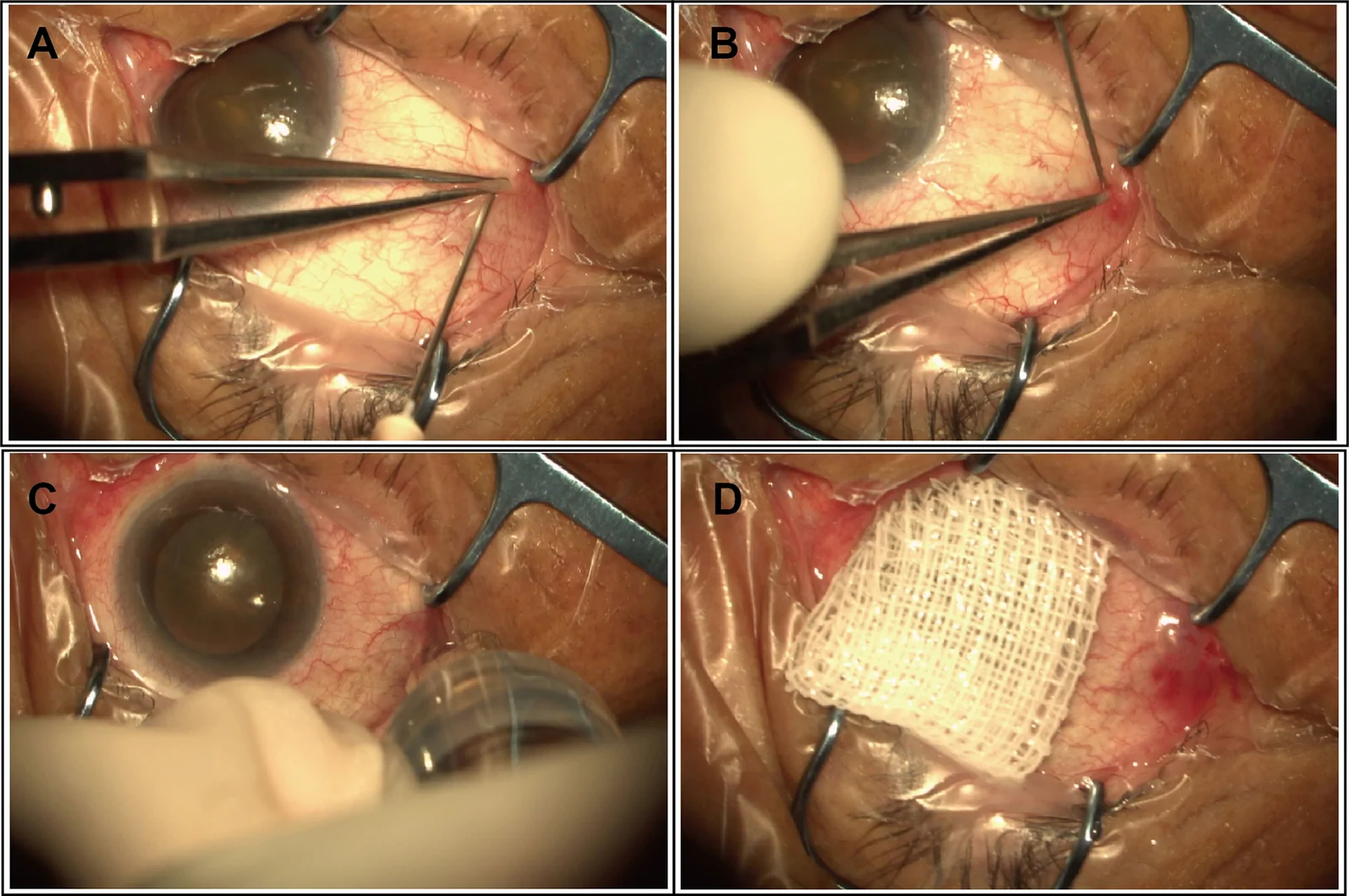
Surgical procedure of retrobulbar block via the fornix conjunctival approach (A) Subconjunctival injection of 0.1 mL local anesthetic in the inferotemporal conjunctival fornix. (B) Insertion of the curved blunt-tip needle via the fornix conjunctival approach. (C) Retrobulbar injection of 2 mL of ropivacaine into the muscle cone. (D) Application of a saline-moistened pad to protect the cornea.

**Figure 3:**
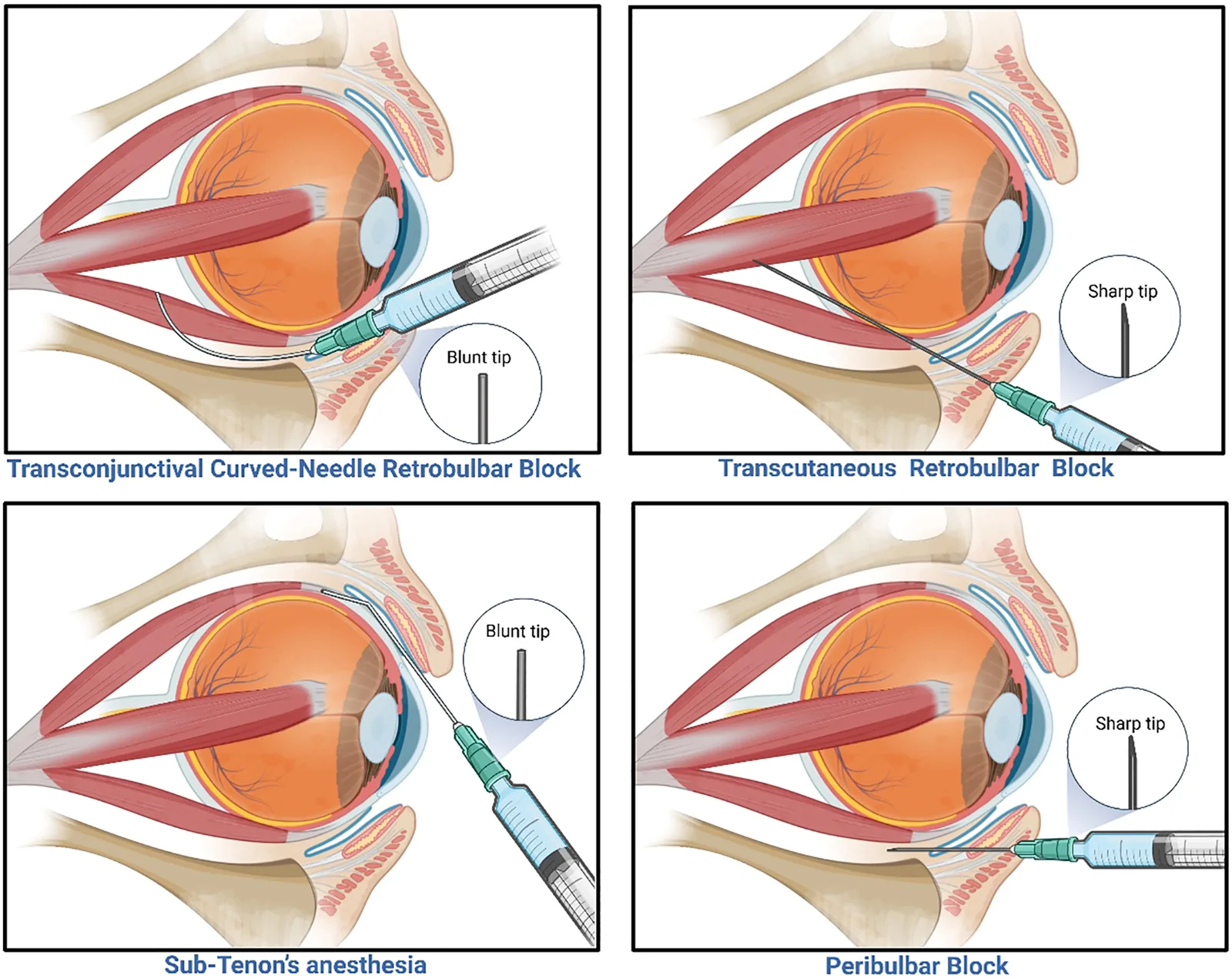
Schematic comparison of ocular anesthesia techniques This figure illustrates the anatomical approaches, needle tip characteristics, and spatial relationships of four common ocular anesthesia methods, including the *trans*-fornix approach and other mainstream techniques.

## Results

### Study participants

A total of 196 patients were enrolled and randomized in this study. The short-duration cohort included 103 patients, with 52 in subgroup A1 and 51 in subgroup B1, while the long-duration cohort comprised 93 patients, with 47 in subgroup A2 and 46 in subgroup B2 (Figure 4).

**Figure 4:**
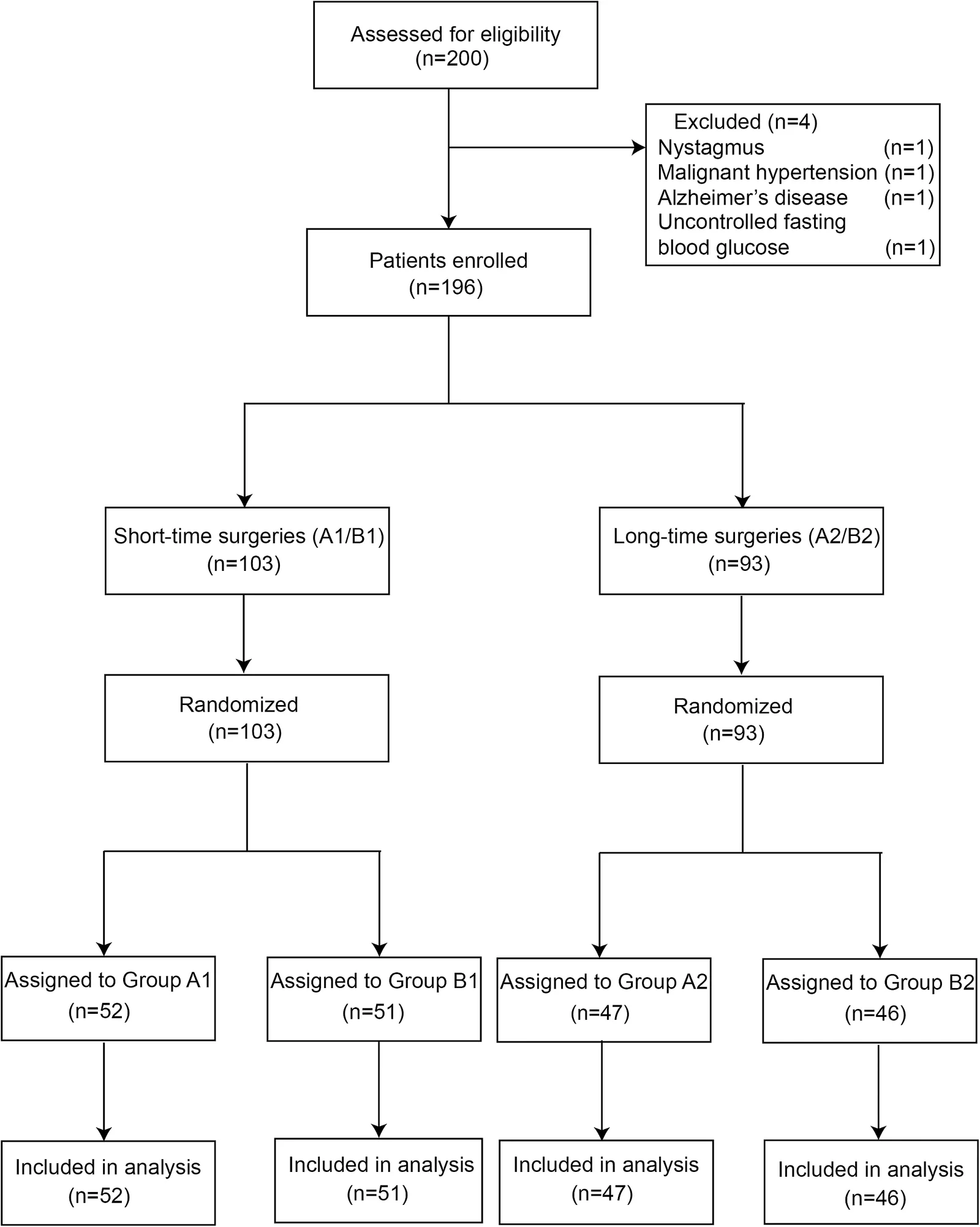
CONSORT flow diagram of the study The diagram illustrates the flow of participants through the trial, including enrollment, categorization, randomization, allocation, and analysis. A total of 196 patients were enrolled and stratified by estimated surgery duration into short-duration (*n* = 103) and long-duration (*n* = 93) cohorts. Subsequently, patients were randomized into either the control group (group A) or the experimental group (group B), resulting in four subgroups: A1 and B1 (short-duration), and A2 and B2 (long-duration). All randomized patients were included in the final analysis.

The baseline demographic and ocular characteristics, including age, sex, logMAR visual acuity, IOP, AL, and ECD, are summarized in Tables 1 and 2. Statistical analysis revealed no significant differences between the control and experimental groups in either the short- or long-duration cohorts (all *p* > 0.05). These results indicate that the groups were comparable at baseline.

**Table 1:** Baseline information of the A1/B1 patients

	A1 (*n* = 52)	B1 (*n* = 51)	*p* value
Age	63.55 ± 11.23	63.82 ± 13.25	0.9135
Sex (M/F)	22/30	22/29	>0.9999
logMAR	1.43 ± 1.06	1.60 ± 0.94	0.4290
IOP	16.61 ± 6.04	15.92 ± 4.36	0.4849
AL	25.20 ± 3.28	24.98 ± 3.33	0.4507
ECD	2,577.04 ± 376.09	2,602.15 ± 499.98	0.8477
Surgery time	17.06 ± 1.83	17.58 ± 1.78	0.1657
Procedure, *n*	–	–	>0.9999
ERM peeling	16	16	–
Oil removal	19	18	–
IOL fixation	15	15	–
GDD implantation	2	2	–

Data are shown in the form of mean ± standard deviation, or as the number of patients. Continuous variables were compared using the independent-samples *t* test. The distribution of surgical procedures was compared using Fisher’s exact test. Sex (M/F): Male/Female. logMAR: logarithm of the minimum angle of resolution. IOP: intraocular pressure. AL: axial length. ECD: endothelial cell density. ERM peeling: epiretinal membrane peeling. Oil removal: silicone oil removal. IOL fixation: ciliary sulcus suture fixation of an intraocular lens. GDD implantation: glaucoma drainage device implantation.

**Table 2:** Baseline information of the A2/B2 patients

	A2 (*n* = 47)	B2 (*n* = 46)	*p* value
Age	58.51 ± 12.78	59.20 ± 12.90	0.7997
Sex (M/F)	28/19	26/20	0.8349
logMAR	1.84 ± 1.04	1.92 ± 0.99	0.6861
IOP	14.09 ± 3.35	15.31 ± 3.85	0.1109
AL	23.98 ± 2.59	23.88 ± 2.75	0.5541
ECD	2,624.98 ± 374.93	2,676.46 ± 335.65	0.6021
Surgery Time	46.91 ± 11.38	48.70 ± 9.30	0.3487
Procedure, n	–	–	>0.9999
Macular hole	4	3	–
Retinal detachment	20	21	–
PDR	23	22	–

Data are shown in the form of mean ± standard deviation, or as the number of patients. Continuous variables were compared using the independent-samples *t* test. The distribution of surgical procedures was compared using Fisher’s exact test. Sex (M/F): Male/Female. logMAR: logarithm of the minimum angle of resolution. IOP: intraocular pressure. AL: axial length. ECD: endothelial cell density. Macular hole: macular hole surgery with tamponade. Retinal detachment: retinal detachment repair. PDR: pars plana vitrectomy for stage V or VI proliferative diabetic retinopathy.

### VAS-based pain grades for patients

As shown in Table 3, group B demonstrated significantly lower VAS-based pain grades during anesthetic injection in both the short-duration (1.33 ± 0.47 vs. 1.94 ± 0.69, *p* < 0.0001) and long-duration cohorts (1.35 ± 0.48 vs. 1.87 ± 0.61, *p* < 0.0001). While pain grades during incision and manipulation were comparable between groups (*p* > 0.05), group B showed a marked advantage during scleral indentation in the long-duration cohort (1.54 ± 0.77 vs. 2.32 ± 0.95, *p* < 0.0001). Because grades 1 and 2 were prespecified as representing adequate analgesia, the proportion of patients achieving adequate analgesia was additionally compared at each stage and is shown in Table 3. These results suggest that the *trans*-fornix conjunctival approach offers superior analgesia during the injection and indentation phases.

**Table 3:** VAS-based pain grades for patients at different stages

	Stages	Group A	Group B	*p* value
A1/B1 group	–	A1 (*n* = 52)	B1 (*n* = 51)	–
	Injection	1.94 ± 0.69 14/27/11/0 41/52 (78.8%)	1.33 ± 0.47 34/17/0/0 51/51 (100%)	<0.0001∗∗∗∗ 0.0005
	Incision creation	1.21 ± 0.41 41/11/0/0	1.16 ± 0.36 43/8/0/0	0.6127
	Intraoperative	1.19 ± 0.40 42/10/0/0	1.14 ± 0.34 44/7/0/0	0.5969
A2/B2 Group	–	A2 (*n* = 47)	B2 (*n* = 46)	–
	Injection	1.87 ± 0.61 12/29/6/0 41/47 (87.2%)	1.35 ± 0.48 30/16/0/0 46/46 (100%)	<0.0001∗∗∗∗ 0.0264
	Incision creation	1.23 ± 0.42 36/11/0/0	1.15 ± 0.36 39/7/0/0	0.4320
	Intraoperative	1.15 ± 0.36 40/7/0/0	1.15 ± 0.36 39/7/0/0	>0.9999
	Scleral indentation	2.32 ± 0.95 9/21/10/7 30/47 (63.8%)	1.54 ± 0.77 28/12/5/1 40/46 (87.0%)	<0.0001∗∗∗∗ 0.0153

Data are presented as mean ± standard deviation (SD), the number of patients in VAS-based pain grades 1/2/3/4, and, where the groups differed, the proportion achieving adequate analgesia (grades 1–2), a prespecified definition. Ordinal comparisons used the Mann-Whitney U test. Proportions were compared using Fisher’s exact test. Adequate analgesia was achieved in all patients of both groups at incision creation and intraoperative manipulation. The between-group difference in adequate analgesia was +21.2% (95% CI 10.1 to 32.3) at injection in the short-duration cohort, +12.8% (3.2–22.3) at injection in the long-duration cohort, and +23.1% (6.3–40.0) at scleral indentation. ∗∗∗∗, *p* < 0.0001. *n* refers to the number of patients.

### Brahma movement score and complications

Table 4 summarizes the ocular motility scores and anesthesia-related complications. The efficacy of ocular motility was evaluated using the Brahma scoring system,^7^ in which lower scores indicate better movement control. Overall, group B achieved significantly better ocular akinesia compared to group A (mean Brahma score: 1.07 ± 0.58 vs. 1.29 ± 0.69, *p* = 0.0157). In the short-duration cohort, subgroup B1 exhibited a significantly lower Brahma score than subgroup A1 (*p* = 0.0437). Although the difference between the long-duration subgroups (A2 vs. B2) did not reach statistical significance (*p* = 0.1728), a favorable numerical trend toward improved akinesia was observed with the *trans*-fornix approach. Regarding anesthesia-related complications, group A reported 8 cases of “raccoon eyes” (subcutaneous ecchymosis) and 2 cases of periorbital hemorrhage. No such complications were observed in group B. Because these events were infrequent and the trial was not powered to compare complication rates, this difference should be interpreted descriptively.

**Table 4:** Brahma movement score and complications

	Stages	Group A	Group B	*p* value
A1/B1 group	–	A1 (*n* = 52)	B1 (*n* = 51)	–
	Brahma movement score	1.25 ± 0.65	1.00 ± 0.59	0.0437∗
	Raccoon eyes	4/52	0/51	–
	Periorbital hemorrhage	2/52	0/51	–
	Supplemental anesthesia	0/52	0/51	–
A2/B2 group	–	A2 (*n* = 47)	B2 (*n* = 46)	–
	Brahma movement score	1.34 ± 0.72	1.15 ± 0.55	0.1728
	Raccoon eyes	4/47	0/46	–
	Periorbital hemorrhage	0/47	0/46	–
	Supplemental anesthesia	7/47	1/46	–
A/B group	–	A (*n* = 99)	B (*n* = 97)	–
	Brahma movement score	1.29 ± 0.69	1.07 ± 0.58	0.0157∗

Data are presented as mean ± standard deviation (SD). Brahma scores were compared using the Mann-Whitney U test, and *n* refers to the number of patients. Complication and supplemental anesthesia data are presented descriptively as event counts and denominators; owing to the small number of events, no formal statistical comparison was performed (indicated by “-”). ∗, *p* < 0.05.

## Discussion

The design of our technique is based on precise ocular anatomy. Generally, the average axial length of a normal globe is approximately 24 mm, and the lateral conjunctival fornix is located about 12 mm posterior to the corneal limbus. Within the intraconal space, the ciliary ganglion lies approximately 1 cm anterior to the common tendinous ring, positioned between the optic nerve and the lateral rectus muscle. Retrobulbar block anesthesia involves the injection of a local anesthetic into the muscle cone posterior to the globe, thereby anesthetizing the ciliary ganglion, oculomotor nerve, and abducens nerve. This allows the anesthetic to diffuse to the ciliary ganglion as well as to branches of the oculomotor and abducens nerves. Therefore, it can provide stable surgical eye positioning and create the conditions necessary for painless intraocular surgery.

The traditional transcutaneous retrobulbar block is generally performed as a blind procedure. The successful delivery of the anesthetic into the muscle cone is influenced by multiple factors, including needle insertion depth, operator’s experience, and the physician’s concern about using sharp needles. Numerous complications have been reported, including globe perforation^8^ and retrobulbar hemorrhage.^9^ To reduce the risk of hemorrhage, external ocular compression is routinely applied after injection. However, inappropriate compression may lead to complications such as retinal or optic nerve ischemia and postoperative ptosis.^10^ Inadequate puncture technique can result in intraocular hemorrhage and secondary retinal detachment,^11^ whereas excessive needle advancement markedly increases the risk of optic nerve injury. Although rare, brainstem anesthesia represents a severe and potentially life-threatening complication.^12^ This may occur if the anesthetic is inadvertently injected into the ophthalmic artery or penetrates the optic nerve sheath, allowing spread into the cerebrospinal fluid and subsequent involvement of the central nervous system.

Regarding the initial stage of needle insertion, Table 3 reveals a statistically significant difference in VAS-based pain grades between the two groups. Patients in group A reported significantly higher pain scores compared to those in group B. This difference is likely attributed to the use of a larger-gauge (25G) needle in group A. Although a finer needle (27G) might theoretically reduce insertion pain, disposable needles of adequate length for the transcutaneous approach are not widely available in our region. Moreover, excessively long and fine needles may lack sufficient rigidity for accurate directional control, potentially increasing the risk of globe perforation. In contrast, the fornix conjunctiva is loosely attached to the underlying tissue, providing an anatomical plane that facilitates smooth needle advancement. Additionally, no statistically significant difference was observed between the two groups in terms of pain during incision creation or overall intraoperative manipulation. This indicates that for standard surgical steps and shorter procedures, both techniques using an injection volume of approximately 2.0 mL can provide adequate and comparable analgesia. Furthermore, there is a difference in the long-duration surgery subgroups regarding the need for supplemental anesthesia (Table 4). In group A2, seven cases required intraoperative supplementation, whereas only one case in group B2 required additional anesthesia. This increased need for supplementation in group A is partly correlated with the pain experienced during scleral indentation, corresponding to the fourth procedural stage analyzed in Table 3.

Analysis of postoperative ocular motility in Table 4 demonstrates that, with the same anesthetic volume, the fornix conjunctival retrobulbar block achieved superior ocular akinesia. This enhanced efficacy is likely due to more accurate intraconal deposition of the anesthetic facilitated by this approach. Achieving excellent intraoperative akinesia is crucial for performing precise procedures during PPV, such as macular hole tamponade, retinotomy, and membrane peeling. Furthermore, effective globe immobilization may also contribute to reducing discomfort associated with traction on the extraocular muscles. Regarding the analysis of anesthesia-related complications in Table 4, no severe complications, such as retrobulbar hemorrhage, optic nerve injury, globe perforation, or brainstem anesthesia, were observed in either group. The complications in group A were primarily limited to periorbital hemorrhage and “raccoon eyes” observed one week postoperatively. These were attributed to the use of a sharp-tipped needle, which increased the possibility of puncturing the abundant subcutaneous and intraorbital vasculature.

In conclusion, the retrobulbar block via the fornix conjunctival approach with a curved blunt needle demonstrates several distinct advantages. (1) The entire procedure is done under a microscope, allowing precise needle placement and facilitating a shorter learning curve. Combined with topical anesthesia, this approach significantly reduces injection-related discomfort compared with the conventional transcutaneous technique, thereby enhancing overall patient experience. (2) The anatomical positioning is more controlled and reproducible, ensuring reliable anesthesia. The curved blunt needle follows the scleral curvature directly into the muscle cone. This precise injection effectively blocks the ciliary ganglion, oculomotor nerve, and abducens nerve, simultaneously achieving both ocular analgesia and extraocular muscle akinesia. Additionally, the design characteristics of the device may further reduce the risk of severe complications. (3) The needle features a blunt tip and a shortened shaft length of 20 mm, compared with the conventional 38 mm needle, which may decrease the likelihood of deep orbital penetration and optic nerve injury. Furthermore, the blunt tip design may reduce the risk of orbital hemorrhage and inadvertent injury to the extraocular muscles. (4) The anesthetic dosage is significantly reduced, eliminating the need for ocular compression. Due to the precise injection directly into the muscle cone, only 1.5–2.0 mL of local anesthetic is required, compared to the 3.5–5.5 mL typically needed for traditional retrobulbar blocks.^13^^,^^14^ This reduction in volume may decrease orbital pressure elevation and minimize the risk of systemic anesthetic toxicity. (5) Intraoperative supplementation is reduced and can be performed safely. If additional anesthesia is required during surgery, it can be administered directly through the original puncture site without removing the eyelid speculum or interrupting the procedure. This feature is particularly beneficial for cases with open corneal wounds, as it enhances surgical safety while maintaining procedural continuity and efficiency. (6) This technique is fundamentally distinct from sub-Tenon’s anesthesia. Sub-Tenon’s anesthesia involves injecting the anesthetic into the potential space between the sclera and Tenon’s capsule, relying on diffusion for its effect. Consequently, complete blockade of intraconal neural structures may be inconsistent. In contrast, our technique delivers the anesthetic directly into the muscle cone, providing more reliable neural blockade without interfering with the surgical field. The two approaches therefore differ in kind rather than in degree. Sub-Tenon’s anesthesia is a form of local infiltration, whereas the present technique is a nerve block delivered within the muscle cone. The difference is also evident during surgery. After injection with the present technique, only mild conjunctival edema is seen around the needle tunnel, reflecting limited seepage of anesthetic into the adjacent tissue planes. After sub-Tenon’s anesthesia, the anesthetic accumulates in the space between the sclera and Tenon’s capsule, and extensive subconjunctival edema of varying degree is commonly observed.

This comparison applies to conventional sub-Tenon’s infiltration. The idea of delivering anesthetic to the retrobulbar or intraconal space through a trans-Tenon’s route has been described previously, including by Sugimoto et al. and by Xu et al. The contribution of the present study is therefore not the anatomical concept itself, but a simpler and more reproducible clinical method. A single 20-mm 27-gauge curved blunt-tip needle is introduced through the conjunctival fornix in one pass and advanced into the muscle cone under the operating microscope. No conjunctival or Tenon’s capsule incision is made, no exchange between a cannula and a needle is required, and no sharp needle is advanced within the orbit. The insertion depth is adjusted to the individual axial length, and the block is completed with approximately 2.0 mL of anesthetic without routine ocular compression afterward. The design of the trial is also part of what we hope this work contributes. Rather than assessing pain as a single global measure, we stratified patients by the expected duration of surgery and prespecified four assessment points covering anesthetic injection, incision creation, intraoperative manipulation and scleral indentation. This allowed us to identify where the advantage of the technique actually lies, namely lower injection-related pain, better ocular akinesia, and a reduced need for supplemental anesthesia during prolonged vitreoretinal procedures.

### Limitations of the study

This study has several limitations. First, although the sample size was adequate for the primary outcomes, a larger, multicenter cohort would further strengthen the generalizability of our findings. The trial was powered for the primary analgesia outcome and was not powered to detect between-group differences in rare anesthesia-related complications. The absence of severe complications in either group therefore suggests a favorable safety profile rather than establishing a reduction in risk. Second, the follow-up period was relatively short. While the present study focused on acute anesthesia-related complications, extended follow-up would be necessary to comprehensively assess long-term safety and functional outcomes. Third, the VAS-based pain grade is a subjective parameter that can be influenced by individual pain tolerance and perioperative anxiety, although we attempted to minimize bias by masking patients and outcome assessors to group allocation and standardized perioperative management. Fourth, group B received an additional 0.1 mL subconjunctival injection before the blunt-tip needle was inserted. This step is part of the technique itself, so the reduction in injection-related pain reflects the whole approach rather than the needle design alone. Fifth, the intraconal position of the needle tip was judged clinically and was not confirmed by imaging. Finally, further prospective studies are warranted to evaluate the learning curve associated with this technique, particularly among junior ophthalmologists, in order to determine its reproducibility and training feasibility in routine clinical practice.

## Resource availability

### Lead contact

Further information and requests for resources should be directed to and will be fulfilled by the lead contact, Bing Chen (chenbing751@126.com).

### Materials availability

This study did not generate new unique reagents. The curved blunt-tip needle evaluated in this study is commercially available from Nanjing ChuangKeDa Medical Instrument Co., Ltd. (Jiangsu, China).

### Data and code availability

De-identified individual participant data reported in this study will be shared by the lead contact upon request. To protect participant privacy, the data are not deposited in a public repository, and requests will be reviewed in accordance with the ethics approval for this study. This study does not report original code. Any additional information required to reanalyze the data reported in this study is available from the lead contact upon request.

## Acknowledgments

No specific funding was received for this study.

## Author contributions

R.C. conducted the literature search, designed the study protocol, processed the data, and wrote the manuscript. T.X. and C.C. processed the data and wrote the manuscript. B.C. designed the study protocol, performed all retrobulbar blocks, and provided funding for the study. K.H. critically revised the manuscript and guided the revision process. K.L., G.S., C.X., M.Y., and R.C. collected the data and critically reviewed the manuscript. All authors read and approved the final manuscript.

## Declaration of interests

The authors declare no competing interests.

## STAR★Methods

### Key resources table

**Table:** 

REAGENT or RESOURCE	SOURCE	IDENTIFIER
**Chemicals, peptides, and recombinant proteins**		

Ropivacaine hydrochloride injection, 1% (10 mL: 100 mg)	Yichang Humanwell Pharmaceutical Co., Ltd., Hubei, China	N/A
Oxybuprocaine hydrochloride eye drops, 0.1%	Santen Pharmaceutical Co., Ltd., Osaka, Japan	N/A

**Software and algorithms**		

GraphPad Prism version 10.6.0	GraphPad Software, Boston, MA, USA	RRID:SCR_002798
G∗Power version 3.1	Heinrich Heine University Dusseldorf, Germany	RRID:SCR_013726

**Other**		

27-gauge, 20-mm curved blunt-tip needle	Nanjing ChuangKeDa Medical Instrument Co., Ltd., Jiangsu, China	N/A
25-gauge retrobulbar needle	Changzhou Medical Device Factory, Jiangsu, China	N/A

### Experimental model and study participant details

#### Study setting and participants

This prospective, multicenter, participant- and assessor-masked randomized clinical trial was conducted at the Changzhou Branch Hospital of Xiamen Eye Center Affiliated to Xiamen University (Purui Eye Hospital) and Gaoyou Bright Eye Hospital between February 2025 and December 2025. Of the 196 participants, 158 were enrolled at Purui Eye Hospital and 38 at Gaoyou Bright Eye Hospital. All retrobulbar blocks in both groups and at both centers were performed by the same experienced ophthalmologist (Bing Chen), following an identical standardized protocol for the entry site, the insertion depth, the injection volume and the injection rate. Because the two techniques differ in entry site and instrument, the operator could not be masked to group allocation. Patients were not informed of their group allocation. Pain grades and Brahma scores were recorded by an independent researcher who was also masked to group allocation. The study was conducted in accordance with the tenets of the Declaration of Helsinki and was approved by the Medical Ethics Review Committee for Clinical Research at Xiamen Eye Center, Xiamen University (approval number: XMYKZX-KY-2024-083). Written informed consent was obtained from all participants prior to enrollment. The trial was prospectively registered with the Chinese Clinical Trial Registry (ChiCTR2500097493) on February 19, 2025, and the first patient was enrolled on February 24, 2025 (https://www.chictr.org.cn/bin/project/edit?pid=260810).

#### Inclusion and exclusion criteria

Inclusion criteria: (1) Patients aged 18 to 82 years undergoing one of the following surgical procedures: pars plana vitrectomy (PPV), silicone oil removal, ciliary sulcus suture fixation of intraocular lens (IOLs), or glaucoma drainage device implantation; (2) Eligible participants demonstrated no systemic contraindications following preoperative evaluation, were capable of cooperating during local anesthesia; (3) Patients who voluntarily agreed to participate in the clinical trial and provided written informed consent.

Exclusion criteria: (1) Patients unable to cooperate with surgery under local anesthesia; (2) Patients with a known history of allergy to ropivacaine; (3) Children, or women who were pregnant or lactating; (4) Patients with communication barriers or cognitive impairments preventing accurate VAS assessment; (5) Patients with nystagmus, malignant hypertension, or other severe comorbidities.

#### Ethics approval and consent

This study was conducted in accordance with the Declaration of Helsinki and was approved by the Medical Ethics Review Committee for Clinical Research at Xiamen Eye Center, Xiamen University (approval number: XMYKZX-KY-2024-083).

Written informed consent was obtained from all participants prior to enrollment.

#### Participant characteristics

Participants were aged 18 to 82 years, and both female and male patients were enrolled. The age and sex distributions of each subgroup are reported in Tables 1 and 2 and did not differ between groups. All participants were of Han Chinese ethnicity. The influence of sex on the outcomes was not analyzed, because the trial was not powered for sex-stratified comparisons. This may limit the generalizability of the findings.

### Method details

#### Randomization and grouping

Patients were stratified preoperatively according to the estimated surgical duration into two strata: short-duration procedures (≤20 min) and long-duration procedures (>20 min). Stratification was performed based on the surgeon’s preoperative assessment of surgical complexity.

Within each stratum, patients were randomly assigned in a 1:1 ratio to receive either the traditional transcutaneous retrobulbar block (**Group A**) or the *trans*-fornix retrobulbar block (**Group B**). The randomization sequence for each stratum was computer-generated using block randomization to ensure balanced allocation. Allocation concealment was maintained using sealed, sequentially numbered, opaque envelopes prepared by an independent study coordinator who was not involved in patient recruitment or outcome assessment. The envelopes were opened immediately prior to anesthesia administration. Accordingly, four subgroups were formed: A1 and B1 (short-duration procedures), and A2 and B2 (long-duration procedures). Subgroups A1 and B1 included relatively short procedures, such as PPV for epiretinal membrane (ERM) peeling, silicone oil removal, ciliary sulcus suture fixation of IOLs, and glaucoma drainage device implantation. Subgroups A2 and B2 comprised prolonged, complex interventions, including macular hole surgery with tamponade, retinal detachment repair, and PPV for stage V or VI proliferative diabetic retinopathy (PDR) that required scleral indentation for peripheral vitreous base removal.

#### Anesthesia procedure

In Group A (the control group), the retrobulbar block anesthesia was performed using the traditional transcutaneous approach via a blind technique. Topical anesthesia was first induced by instilling 0.1% oxybuprocaine (Santen Pharmaceutical Co., Ltd., Japan) into the conjunctival sac twice. The puncture site was located at the junction of the lateral one-third and medial two-thirds of the inferior orbital rim. During the procedure, the patient was instructed to maintain a superonasal gaze. A 25-gauge retrobulbar needle attached to a 5-mL disposable syringe (Changzhou Medical Device Factory, Jiangsu, China) was used. The needle was initially inserted posteriorly, parallel to the orbital floor, until it reached the equator of the globe. Subsequently, it was redirected toward the retrobulbar space. It was slowly advanced between the lateral rectus and inferior rectus muscles until entry into the muscle cone was perceived. The needle bevel faced the globe to minimize the risk of perforation, and insertion depth was strictly limited to 35 mm. After negative aspiration confirmed no blood reflux, 2.0 mL of 1% ropivacaine hydrochloride injection (10 mL: 100 mg) was slowly injected. The needle was then withdrawn, and external compression was applied to the globe for 5 min. Finally, the surgery began following routine disinfection and draping.

In Group B (the experimental group), the retrobulbar block anesthesia was performed via the fornix conjunctival approach using a curved blunt-tip needle (Figure 1). Following routine disinfection and draping, the procedure was applied using an operating microscope.

Topical anesthesia was induced by instilling 0.1% oxybuprocaine (Santen Pharmaceutical Co., Ltd., Japan) into the conjunctival sac twice. The patient was instructed to gaze superonasally. Initially, a subconjunctival injection of approximately 0.1 mL of local anesthetic was administered with a 30-gauge needle in the inferotemporal conjunctival fornix (Figure 2A). Subsequently, a 20-mm 27-gauge curved blunt-tip needle (Nanjing ChuangKeDa Medical Instrument Co., Ltd., Jiangsu, China) was inserted, either passing through the initial puncture site or directly through the conjunctiva (Figure 2B). The needle was advanced approximately 5 mm parallel to the sclera posterior to the equator. The patient was then instructed to gaze directly upward, and the needle was advanced along the scleral curvature until it entered the muscle cone. The depth of needle insertion was determined based on the preoperative axial length. In eyes with high myopia and long axial length, the needle shaft was advanced fully into the conjunctival entry site, whereas in eyes with a normal or short axial length, approximately 2 mm of the needle shaft was left outside the conjunctival entry site. 2.0 mL of 1% ropivacaine hydrochloride injection (10 mL: 100 mg) was slowly injected (Figure 2C). Finally, a moist cotton pad was placed over the cornea to prevent desiccation (Figure 2D), and surgery commenced after a 5-min interval.

A schematic diagram and a video of the four anesthesia methods are provided to illustrate their technical differences (Figure 3; Video S1).

Video S1. Demonstration of the four ocular anesthesia techniques, related to Figure 3

#### Outcome measures

The primary outcome was intraoperative pain, assessed using a four-grade scale derived from the visual analog scale (VAS). Secondary outcomes were ocular motility (akinesia), the need for supplemental anesthesia, and anesthesia-related complications.

Pain assessment: Intraoperative pain was evaluated using the VAS by an independent researcher who was masked to the anesthesia protocol. Pain scores were recorded at specific surgical milestones stratified by subgroup duration. For patients in subgroups A1 and B1 (procedures lasting ≤20 min), pain was evaluated at three time points: anesthetic injection, incision, and intraoperative manipulation (e.g., pars plana vitrectomy [PPV] or glaucoma drainage device implantation). For patients in subgroups A2 and B2 (procedures lasting >20 min), pain was evaluated at four time points: anesthetic injection, incision, intraoperative manipulation (e.g., vitrectomy, membrane peeling, or fluid-air exchange), and scleral indentation for peripheral vitreous base removal.

The VAS scores (ranging from 0 to 10) were categorized into four grades. Grade 1 (0 points): complete absence of pain; Grade 2 (1–3 points): mild pain with slight discomfort; Grade 3 (4–6 points): moderate pain that was obvious but tolerable; Grade 4 (7–10 points): severe, intolerable pain requiring supplemental anesthetic intervention. The pain grade, rather than the raw VAS score, was the variable recorded at each time point and used in all analyses. Grades 1 and 2 were prespecified as representing adequate analgesia.

Ocular motility assessment: Immediately after anesthesia administration, ocular motility was evaluated by an independent researcher who was masked to both the surgical and anesthesia group assignments. The Brahma scoring system was utilized to assess extraocular movements in four cardinal directions (superior, inferior, medial, and lateral). Ocular motility was graded on a scale of 0–3: 0 = no movement; 1 = minimal movement; 2 = partial movement; and 3 = normal movement.

Anesthesia-related complications, recorded as secondary outcomes, comprised intraoperative globe perforation, retrobulbar hemorrhage, optic nerve injury, and periorbital ecchymosis (“raccoon eyes”) assessed at 1 week postoperatively.

### Quantification and statistical analysis

The sample size was estimated *a priori* using G∗Power version 3.1. Because the *trans*-fornix conjunctival retrobulbar block using a curved blunt-tip needle had not previously been compared directly with the conventional transcutaneous approach, no directly applicable estimates were available on which to base an anticipated effect size. A medium effect size according to Cohen’s convention (w = 0.30) was therefore adopted for the chi-squared test, with a two-sided α of 0.05 and 1° of freedom; the power was set at 0.95 rather than the conventional 0.80–0.90 in order to provide a conservative margin. On this basis, a minimum of 145 participants was required (actual power = 0.951). The planned enrollment was increased to 180 participants (90 per group) to allow for dropout and loss to follow-up. Patients were enrolled consecutively over the predefined recruitment period, and no interim analysis was performed; the final sample of 196 patients therefore exceeded both the calculated minimum and the planned enrollment.

All statistical analyses and data visualization were performed using GraphPad Prism version 10.6.0 (GraphPad Software, USA). The VAS-based pain grade and the Brahma score are ordinal variables and were therefore summarised as mean ± standard deviation together with the number of patients in each grade, and compared between groups using the Mann-Whitney U test. The prespecified proportion of patients achieving adequate analgesia (grades 1–2) was compared using Fisher’s exact test, with the between-group difference reported alongside its 95% confidence interval. Continuous variables were assessed for normality using the Shapiro-Wilk test and compared using the independent-samples *t* test or the Mann-Whitney U test as appropriate. Categorical variables were compared using the chi-squared test or Fisher’s exact test. Because of the small number of events, between-group comparisons of anesthesia-related complications and of the need for supplemental anesthesia are reported descriptively as event counts and denominators, without formal significance testing or effect estimates. A two-sided *p* value <0.05 was considered statistically significant.

The exact value of n and what n represents are stated in the header and footnotes of Tables 1, 2, 3, and 4, where n refers to the number of patients. Pain was compared between the two groups at each procedural stage separately, giving seven comparisons in total. Applying a Bonferroni-corrected threshold of 0.0071, the differences observed at anesthetic injection in both cohorts and at scleral indentation remained statistically significant, and the remaining comparisons remained non-significant. The interpretation of the results is therefore not affected by multiple testing. In Tables 3 and 4, asterisks denote statistical significance (∗*p* < 0.05; ∗∗∗∗*p* < 0.0001), and the statistical test and the number of patients are stated in each table footnote.

### Additional resources

This trial was prospectively registered with the Chinese Clinical Trial Registry on February 19, 2025, under registration number ChiCTR2500097493. The full registry record, including the trial protocol and the prespecified outcome measures, is publicly available. Chinese Clinical Trial Registry record: https://www.chictr.org.cn/bin/project/edit?pid=260810.
